# Insights into the binding mode of MEK type-III inhibitors. A step towards discovering and designing allosteric kinase inhibitors across the human kinome

**DOI:** 10.1371/journal.pone.0179936

**Published:** 2017-06-19

**Authors:** Zheng Zhao, Lei Xie, Philip E. Bourne

**Affiliations:** 1 National Center for Biotechnology Information, National Library of Medicine, National Institute of Health, Bethesda, Maryland, United States of America; 2 Department of Computer Science, Hunter College, The City University of New York, New York, United States of America; 3 The Graduate Center, The City University of New York, New York, United States of America; 4 Office of the Director, National Institutes of Health, Bethesda, Maryland, United States of America; NCI at Frederick, UNITED STATES

## Abstract

Protein kinases are critical drug targets for treating a large variety of human diseases. Type-III kinase inhibitors have attracted increasing attention as highly selective therapeutics. Thus, understanding the binding mechanism of existing type-III kinase inhibitors provides useful insights into designing new type-III kinase inhibitors. In this work, we have systematically studied the binding mode of MEK-targeted type-III inhibitors using structural systems pharmacology and molecular dynamics simulation. Our studies provide detailed sequence, structure, interaction-fingerprint, pharmacophore and binding-site information on the binding characteristics of MEK type-III kinase inhibitors. We hypothesize that the helix-folding activation loop is a hallmark allosteric binding site for type-III inhibitors. Subsequently, we screened and predicted allosteric binding sites across the human kinome, suggesting other kinases as potential targets suitable for type-III inhibitors.

## Introduction

Kinases are phosphorylation enzymes that catalyze the transfer of phosphate groups from ATP to specific substrates and are critical in most cellular life processes [1,2]. Abnormal kinase regulation, which leads to signal disruption and cell deregulation, is implicated in many diseases, particularly cancers [3]. Thus, a number of kinase-targeted small molecule inhibitors have been developed that are important in anti-cancer therapy [4]. Through July 2016, 30 small molecule kinase inhibitors [5,6] have been approved by the US Food and Drug Administration (FDA) for the treatment of cancers and other diseases (http://www.fda.gov/) and additional more inhibitors are undergoing clinical trials [7,8].

However, reported off-target toxicities and acquired-mutation resistance [9] require kinase-targeted inhibitors of lower dose and higher specificity. Typically, three types of targeted kinase inhibitors, type-I, type-II and type-III, have been developed [10,11]. Type-I inhibitors are ATP-competitive and occupy the ATP-binding binding pocket, a highly conserved kinase catalytic scaffold with strong binding affinity for ATP. Driven in part by the increased number of diverse protein kinase structures, type-II and type-III inhibitors have also been developed [12]. Type-II inhibitors bind to an extended binding pocket that includes the ATP-binding pocket and the adjacent less-conserved allosteric site across the DFG motif. Although type-II inhibitors occupy larger binding pockets than type-I inhibitors, it has not followed that type-II inhibitors are more selective [13]. However, type-III inhibitors occupy highly specific allosteric sites which provides the opportunity to achieve higher selectivity. To date type-III MEK inhibitors that inhibit MEK1 and/or MEK2 have attracted substantial interest. Dozens of type-III MEK inhibitors have been developed for clinical applications or as molecular probes [14]. Notably, two type-III MEK inhibitors (Trametinib and Cobimetinib) have been approved by the FDA [15,16]. Besides the type-III MEK inhibitors, several type-III inhibitors for other kinases have been reported [17] including the BCR-ABL inhibitors GNF2 and ABL001 [18], the pan-AKT inhibitor MK-2206 [19] and the mutant-selective EGFR allosteric inhibitor EAI045 [20]. In summary, the evidence suggests that type-III inhibitors provide a valuable approach [17,20]. For example, the type-III MEK kinase inhibitor, Cobimetinib (IC50 0.9 nM), overcomes the resistance induced by the BRAF V600E mutation seen in melanoma by inhibiting MEK, which is downstream of BRAF in the BRAF/MEK/ERK pathway [16]. To date, however, there is no systematic means of identifying the preferred characteristics of specific type-III inhibitors [8]. Since existing type-III kinase inhibitors mainly target MEK [17] by understanding the molecular characteristics of type-III MEK inhibitors, the goal is to use that understanding to develop type-III inhibitors more broadly across the human kinome.

In this work we have integrated the structural systems biology strategy and molecular dynamics simulation methods to gain insights into type-III kinase inhibitors and their binding modes with human protein kinases. The structural system biology strategy harnesses multiple omics data resource to compare and discover the gene-level, protein-level and structure-level information on protein-ligand interactions [21]. We have previously applied this strategy to drug design and discovery for the human structural kinome (distinct from the work here) and the Ebola virus proteome [5,22].

Further, with increased computing power and more efficient algorithms, molecular dynamics (MD) simulation is now becoming a routine tool for drug design, accounting for the reality of a flexible target structure and flexible target-drug binding [23]. In this paper we performed detailed MEK-inhibitor interactional fingerprint analysis using the aforementioned methods. This was followed by two MD simulations up to 1.2 μs in an explicit water box to obtain insights into the behavior of MEK as a flexible target, with and without the representative ligand, Cobimetinib [24]. By comparing the structural trajectories between MEK with and without ligand, we determined the structural flexibility and interaction network for type-III inhibitor binding to MEK.

Finally, we studied the structural impact of point mutations, the MEK pharmacophore and the mechanistic understanding of MEK-drug binding. Using these aggregate data as a template we explored the whole human kinome to identify potential new opportunities for type-III inhibition.

## Results

### Binding modes of crystallized ligand-bound MEK complexes

**Fig 1:**
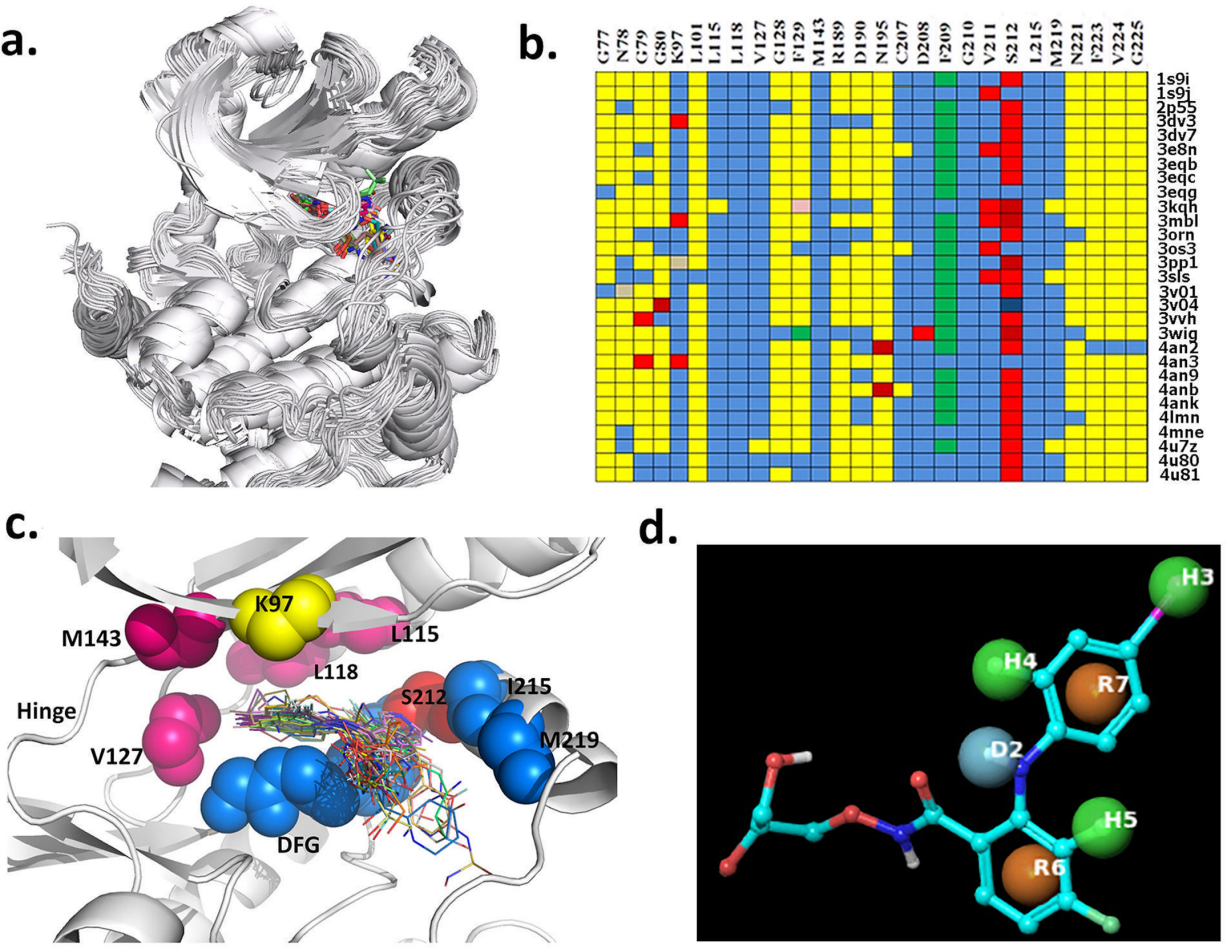
Binding characters of MEK-ligand complexes. a) All MEK-ligand complex structures aligned using SMAP. b) Encoding all MEK-ligand interactions. Every row represents the MEK-ligand interaction fingerprint of one complex structure, and every column represents the interactions between the same amino acid in space and the bound ligand in different complex structures. Different colors represent the different types of fingerprint interactions: yellow, no interaction; blue, apolar interaction; red, apolar interaction + hydrogen bond interaction (protein as donor); deep red, hydrogen bond interaction (protein as donor); pink, polar interaction+ aromatic interaction; and grey, apolar interaction + hydrogen bond interaction (protein as acceptor). c) Spatial representation of MEK-ligand interactions. d) Pharmacophore modeling: H, hydrophobic group; R, aromatic ring; D, hydrogen-bond donor.

We obtained the binding characteristics of ligand-bound MEK complexes as shown in Fig 1. Fig 1a illustrates the alignment of the 29 catalytic kinase domains of MEK bound to the type III inhibitors shown in the same allosteric binding site. We calculated the detailed interactions between MEK and the ligand using the function-site interaction fingerprint (Fs-IFP) method (Fig 1b). The highly conserved interactions between the respective ligands and MEK include K97, L115, L118, V127, M143, C^207^DFGVS^212^, I215 and M219 (Fig 1b and 1c). These conserved interactions can be divided into three spatial regions.

**Fig 2:**
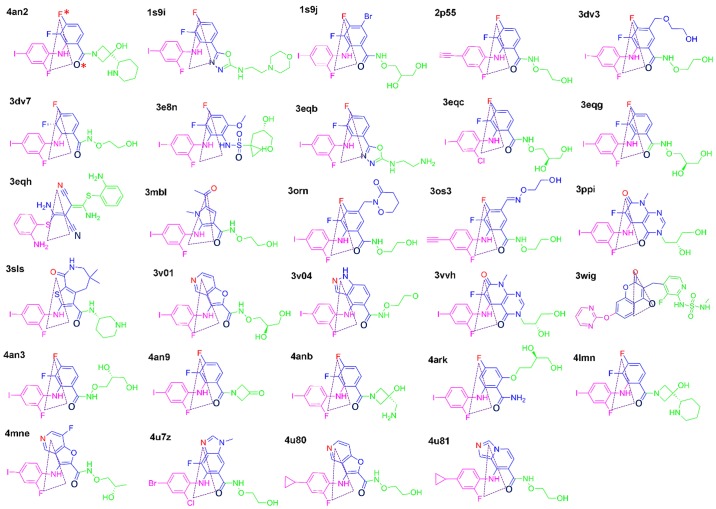
Ligands from the 29 MEK-ligand complex structures. The triangles highlight the conserved structure-activity relationships (SARs) that characterize the MEK Type-III inhibitors in 3D space.

The first region is the hydrophobic sub-pocket consisting of L115, L118, V127 and M143 as shown in purple in Fig 1c. All interactions are apolar (Fig 1b). Correspondingly, all ligands have hydrophobic groups that can be accommodated in the sub-pocket. For example, Cobimetinib has a 2-fluoro-4-iodoanilino fragment, as shown in purple in Fig 2 (4an2), which is a well-known hydrophobic pocket binder and accommodates the hydrophobic sub-pocket with hydrophobic contacts as shown in Fig 1c. Other ligands also have the same or similar fragments (Fig 2, the fragment in purple) so as to achieve the high binding affinity of the conserved sub-pocket. Pharmacophore modeling, as shown in Fig 1d, also revealed similar patterns with three common hydrophobic groups, H3, H4 and R7.

The second region is K97, an important catalytic residue, located at the roof of the binding pocket (Fig 1c, yellow color). K97 has a conserved interaction with the oxygen atom O* (marked in Fig 2, dark blue color) of the respective ligands. The molecular moiety O* is conserved, shown in the same position for other compounds in dark blue, either as an oxygen or nitrogen atom (Fig 2, 3eqb and 3eqh). Pharmacophore modeling (Fig 1d) is consistent with one donor-type hydrogen bond, D2.

The third region, consisting of C^207^DFGVS^212^, I215 and M219, forms a loop and a helix that acts like an arm to accommodate the inhibitor in the kinase active site. DFG is directly involved in kinase catalytic activity and is conserved across the human kinome. I215 and M219 are located in the activation loop. Interestingly, in MEK the activation loop folds into a short helix which forms the allosteric site. In most MEK-ligand structures, S212 has a strong interaction with the corresponding ligand (Fig 1b, S212 column, red color) including an apolar and hydrogen-bond interaction. However, in the structures 1s9j, 3egg, 3oss, 4v04 and 4an3, S212 forms only a polar interaction with the corresponding ligand. This conserved interaction is consistent with experiment which shows that S212 plays a key role in phosphorylation by RAF [16]. In this third region all active ligands have one atom (F, N or O. such as F* in Fig 2, 4an2, in red color) that interacts with the backbone of S212. Pharmacophore modeling (Fig 1d, H5 and R6) illustrates that all ligands have common features in their interaction with S212.

Taken together, the aforementioned three regions make major contributions to ligand binding in the allosteric pocket. Summarizing Fig 1d, the hydrophobic heads (H3, H4 and R7) accommodate the hydrophobic sub-pocket and D2 and H5 interact with the roof amino acid K97 and S212 of the loop, respectively. It is expected that an active MEK inhibitor would have these chemical functional groups or similar. Furthermore, in 3D space, these atoms are spatially conserved, as shown by the triangles in Fig 2 for D2, H4 and H5, suggesting that the design of MEK allosteric inhibitors should follow this pharmacophore. Similar conserved spatial requirements have been reported in the design of other allosteric inhibitors [20]. Of course, besides the conserved pharmacophores, different inhibitors are subjected to specific interactions involving other amino acids to achieve selectivity, as shown in Fig 1b. Specifically, the solvent exposed part of the inhibitor, shown in Fig 2 in green, can be substituted by different chemical group or atoms. The pharmacophore has no common features for the solvent exposed part (Fig 1d). These differences reflect different levels of inhibition as illustrated by Rice and coworkers found in the structure-activity relationships while optimizing a series of compounds leading to Cobimetinib [15]. Based on the co-crystal complex structure with Cobimetinib and ACP, an ATP analogue, shown in S1 Fig, the solvent exposed parts of the inhibitor are right next to the γ-phosphate of ATP and an interaction between them is formed. This interaction disturbs the functional conformation of the γ-phosphate of ATP and the substrate being phosphorylated, reducing MEK’s enzymatic activity.

### MEK structural flexibility and insights into the binding mechanism

**Fig 3:**
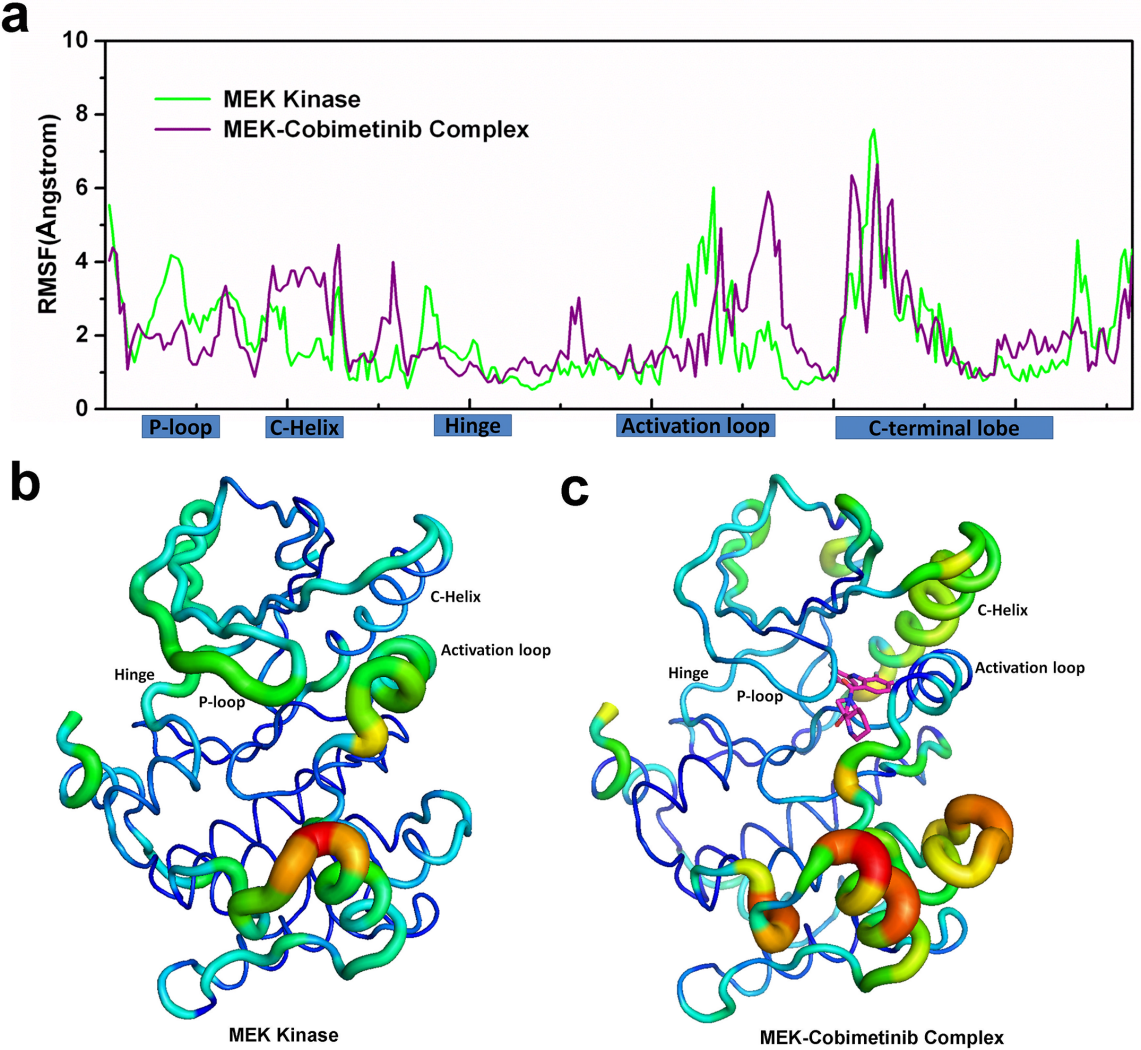
RMSF profiles and PCA projection. a) The RMSF profiles from the last 0.5 μm equilibrated MD trajectories of MEK and the MEK-Cobimetinib complex, respectively. Some secondary structure elements are shown on the abscissa. b) and c) The Cα-atom projection along the first principal component. The displacements are shown as color-coded tubes from blue (small displacement) to orange (large displacement) for (b) MEK and (c) the MEK-Cobimetinib complex.

Beyond studying the binding modes from released PDB structures for MEK, we considered how type-III inhibitors influence MEK structural flexibility. We performed two 0.6 μs MD simulations for MEK kinase with and without the inhibitor bound. The overall Cα-RMSDs of the apo and holo structures are similar (S2 Fig). However, individual Cα-atom fluctuations show significant differences, especially in the ligand-bound regions (Fig 3a), where we compared MEK structural flexibility before and after binding using the two last 0.5 μs equilibrated MD trajectories. For apo MEK the flexibility change mainly comes from the P-loop, the activation loop and the C-terminal lobe as shown in Fig 3a. The corresponding collective motion as inferred from the first principal component of PCA is shown in Fig 3b. As a comparison, in the MEK-Cobimetinib complex, the main fluctuations come from the parts of the C-helix, the C-terminal part of the activation loop and the C-terminal lobe (Fig 3a). The obvious difference before and after binding inhibitor is that the collective motions of the P-loop and activation loop have undergone a substantial reduction and the flexibility of the C-helix has significantly increased in the MEK-Cobimetinib complex compared to apo MEK. Similar to other kinases, the P-loop contributes to conformational flexibility and plays an important role in binding and recognizing phosphoryl moieties [25]. Moreover, this flexible P-loop motif, along with other beta-sheets and helixes, generally form a pocket into which the phosphate groups can insert [26,27]. Like the P-loop, the activation loop shows a similar change in flexibility before and after Cobimetinib binding. Like other kinases, MEK Glu114 within the C-Helix, Lys97 and the DFG peptide form the ATP catalytic center, where the salt-bridge between Glu114 and Lys97 is needed for catalysis. Upon Cobimetinib binding to MEK, the salt-bridge interaction is broken. It may have significant effect on the function of the C-helix [8,28]. In addition, the activation loop forms a short helix in MEK, whereas in most kinases the activation loop is a flexible loop. One hypothesis that follows is that the short helix is important in forming the allosteric binding pocket that accommodates a type-III inhibitor and a necessary consideration in the design of new type-III inhibitors [29].

### Conserved interactions with inhibitors from S212 and K97

**Fig 4:**
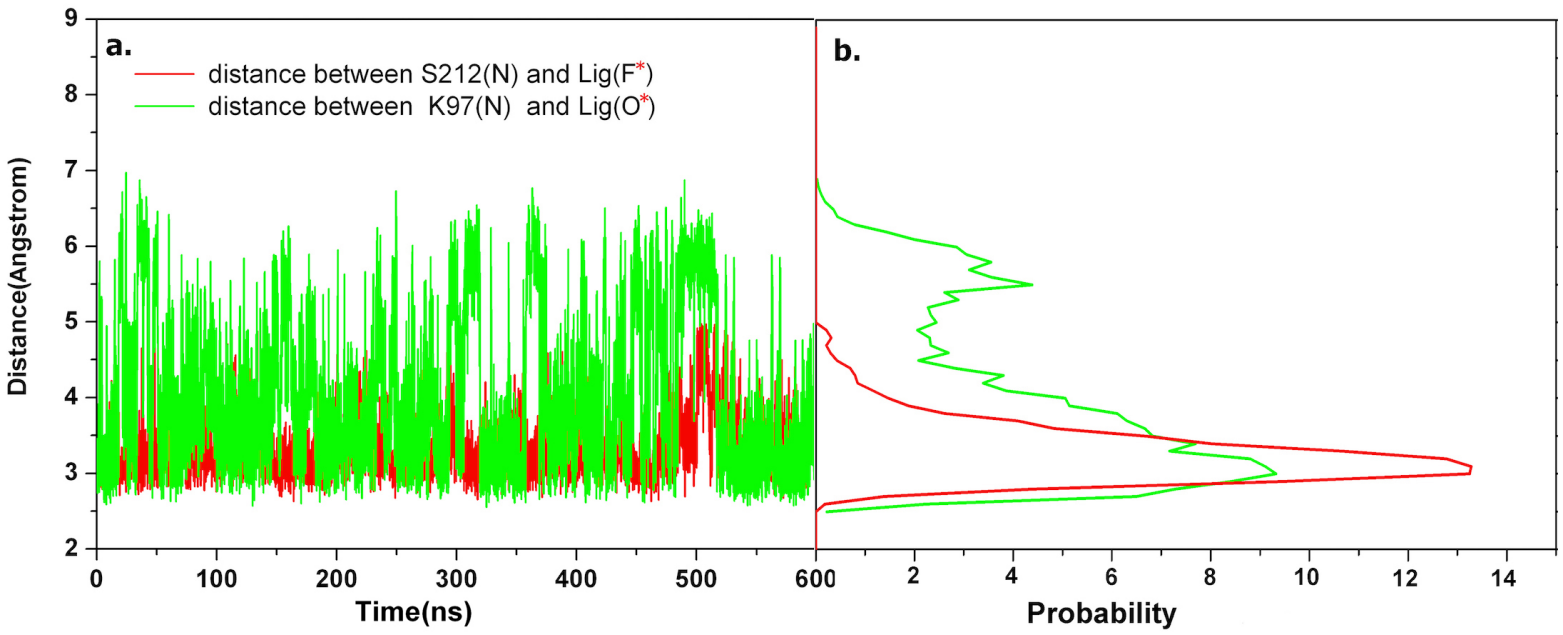
Two conserved interatomic interactions between MEK and the ligand. (a) Interatomic distances for every conformation from the MD trajectory; (b) The probability distribution of interatomic distances.

As aforementioned, the inhibitors derived from PDB MEK structures have a similar core and common functional groups forming a conserved spatial triangular arrangement (Fig 2). Correspondingly, in the MEK-Cobimetinib MD trajectories the conserved interactions between MEK and respective inhibitors were evaluated (Fig 3c). Two key interactions between S212, K97 and Cobimetinib are highlighted here (Fig 4). The interaction between the backbone nitrogen atom of S212 and the F* atom of the inhibitor is shown in red. From the probability distribution (Fig 4b) the center of the peak is at 3.1 Å, which suggests that a hydrogen bond interaction is conserved at all times to maintain the binding affinity and restrain the flexible movement of S212, thereby hindering MEK phosphorylation by RAF. This observed hydrogen bond interaction is in agreement with reported experimental results [16].

The O* atom (Fig 2, 4an2) is another conserved polar atom contributing to the effective binding. As shown in Fig 4 in green, the position of the peak in the probability distribution is at approximately 3.0 Å, which agrees with the distance found in released crystal structures, for example, 4lmn [15,16]. This distance suggests that there is a strong hydrogen-bond interaction between O* of the ligand and the ε-amino group of the lysine (K97), which contributes to the catalytic center [30]. This hydrogen-bond interaction replaces the salt-bridge interaction between the ε-amino group of Lys97 and the Glu114 of the C-Helix. Importantly, blocking the salt-bridge interaction results in an inactive state [31] and increased flexibility of the C-Helix. The O* atom of Cobimetinib, the ε-amino group of Lys97 and the carboxyl group of Asp208, part of DFG, form the pseudo catalytic center and deactivate kinase activity in the MEK/ERK pathway [32]. For other ligands (Fig 2), there is the same oxygen atom or similar nitrogen as O*, which should optionally be retained in future drug design studies.

### Ranking similar binding sites to MEK using SMAP across the structural kinome

To determine all other potential human protein kinases potentially suitable for type-III inhibition, MEK-similar binding pockets screening was performed across the human structural kinome using SMAP [33–35]. Using the SMAP threshold of more than 55% similarity, three crystal structures with binding pockets similar to MEK were found (pdb ids 2yix, 4pp7 and 4wo5). One structure is a P38α kinase [36] and two are BRAF kinases [2]. All three structures contain a small helix within the activation loop. We aligned the sequences of MEK, P38α and BRAF with particular attention to the activation loop. The sequence similarity is not high (S3 Fig; the activation loops are marked with a rectangle). These results lead us to hypothesize that kinases with similar secondary structures in their activation loops have the potential to be inhibited by type-III inhibitors, even though their global sequences do not have high similarity with MEK. Ohren et. al. [29] have also suggested that the helix in the activation loop provides structural insight into designing type-III inhibitors. This then begs the question, what other human protein kinases can potentially form a helix in the activation loop?

### Predicting helix containing activation loops across the human kinome

**Fig 5:**
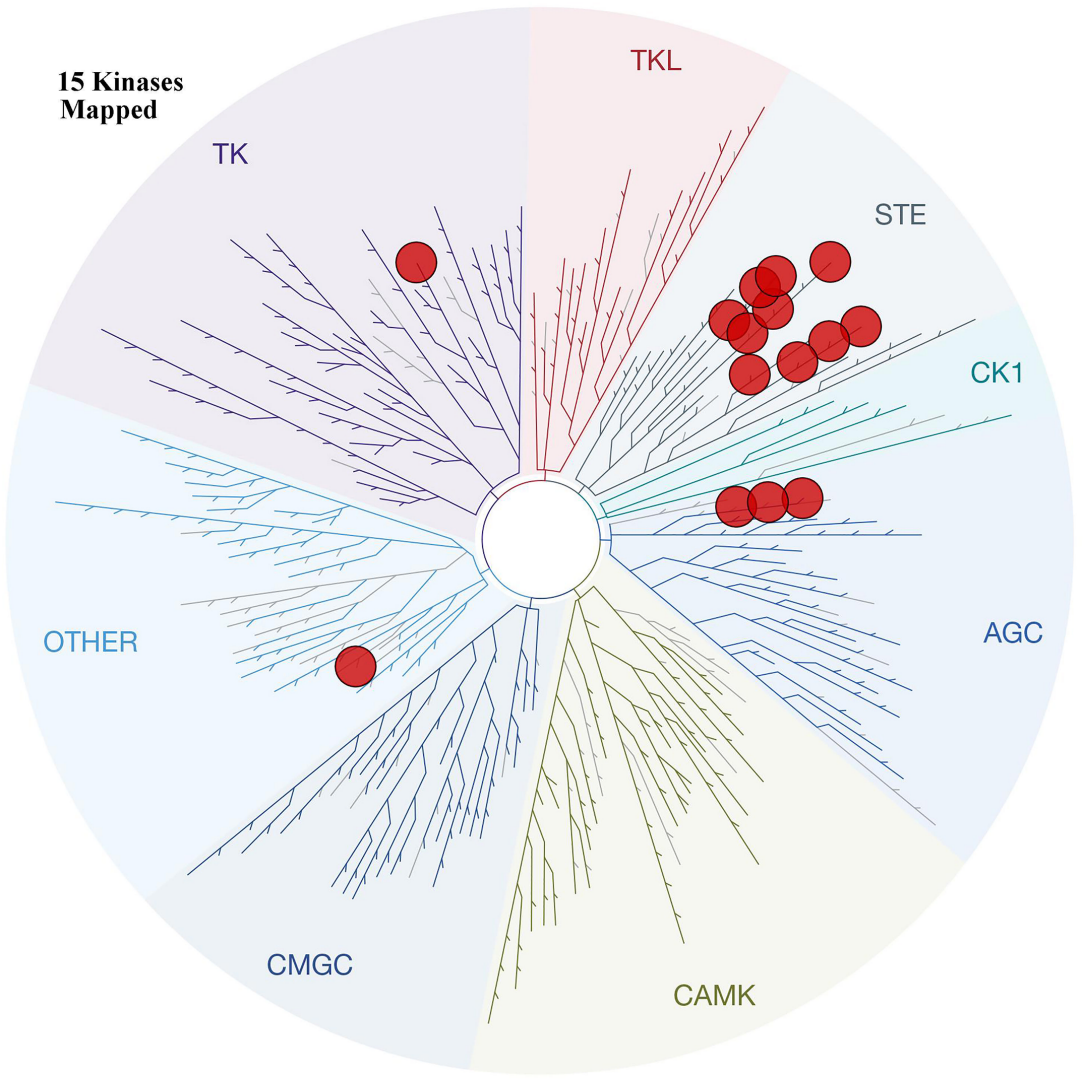
The phylogenetic distribution of the top 15 human protein kinases where a helix was predicted in the activation loop. The figure was generated using TREEspot (www.discoverx.com).

We predicted the secondary structure of the activation loop of all human protein kinases to rank the similarity to the MEK activation loop helix that we suggest is critical for the binding pose of the type-III inhibitor [37]. Based on amino acid sequences of all human protein kinases, we determined the top 15 kinases that have a potential helix within the activation loop [8] (Fig 5 and Table 1). The top 15 kinases are mostly located within the STE group which forms the MAPK cascade. In terms of sequence, their activation loops don’t have high similarity to MEK, yet in terms of secondary structure prediction the activation loops of the 15 kinases may contain a helix. Among them MAP2K4 has been validated by a newly released X-ray structure (pdb id 3alo) [38], in which the crystallized activation loop is shown to contain a helix. If this helix is found in subsequent structures it will be important to consider it in future type-III inhibitor design, partculalrly for the 15 protein kinase targets identified here.

**Table 1:** Top 15 kinases predicted with high confidence to have a helix at part of the activation loop.

Kinases	Sequences at Activation loop	Predicted secondary structure	Confidence of prediction
**TK_JakB__Domain2_JAK3**	DPGVSPAVLSLEMLTDRIPWVA	-----HHHHHHHHH-------	9998535655555404886148
**STE_STE20_KHS_HPK1**	DFGISAQIGATLARRLSFIGTP	------HHHHHHHHH------	9998651226787750368999
**AGC_MAST__MAST3**	DFGLSKIGLMSMATNLYEGHIE	------HHHHHHHHHHH----	9998601144567776216899
**STE_STE20_MSN_ZC1_HGK**	DFGVSAQLDRTVGRRNTFIGTP	----HHHHHHHHHH-------	9963576888764136316899
**STE_STE20_MSN_ZC2_TNIK**	DFGVSAQLDRTVGRRNTFIGTP	----HHHHHHHHHH-------	9963576888764136316899
**STE_STE20_MSN_ZC3_MINK**	DFGVSAQLDRTVGRRNTFIGTP	----HHHHHHHHHH-------	9963576888764136316899
**STE_STE20_KHS_KHS1**	DFGVAAKITATIAKRKSFIGTP	----HHHHHHHHHHH------	9965313789999811125899
**AGC_MAST__MAST1**	DFGLSKMGLMSLTTNLYEGHIE	-----HHHHHHHHHHHH----	9984235488888776425589
**STE_STE7__MAP2K3**	DFGISGYLVDSVAKTMDAGCKP	----HHHHHHHHHHH------	9998146789999832058999
**STE_STE7__MAP2K6**	DFGISGYLVDSVAKTIDAGCKP	----HHHHHHHHHHH------	9998146789999832058999
**AGC_MAST__MAST2**	DFGLSKIGLMSLTTNLYEGHIE	-----HHHHHHHHHHHH----	9984235488888887515589
**STE_STE20_KHS_KHS2**	DFGVSAQITATIAKRKSFIGTP	----HHHHHHHHHHH------	9983799999999803213999
**STE_STE7__MAP2K4**	DFGISGQLVDSIAKTRDAGCRP	-----HHHHHHHHHH------	9987278899999823378999
**STE_STE7__MAP2K5**	DFGVSTQLVNSIAKTYVGTNAY	----HHHHHHHHHHHH-----	9941799999999851688999
**Other_NRBP__NRBP1**	VAPDTINNHVKTCREEQKNLHF	-----HHHHHHHHHHH-----	9966457699999987058999

## Conclusions

Recently approved FDA type-III allosteric protein kinase inhibitors prompted us to consider more efficient and lower-dose kinase inhibitors. Study of the type-III inhibitor-bound binding site provides structural insights into the design of new allosteric inhibitors. Here we study the characteristics of the MEK binding site, the chemical nature of the inhibitors that bind MEK, and the dynamic characteristics and nature of the interaction between protein and inhibitor from MD simulation. Further, based on all 3D kinase structures, we screened for potential allosteric inhibitor-bound binding sites. This revealed that the binding sites of BRAF and P38α were similar to MEK. Finally, based on the distinctive helix character of the MEK activation loop [37] we identified 15 kinases which potentially contain the allosteric site needed to accommodate type-III inhibitors. In summary, our *in silico* analysis furthers our understanding of allosteric protein kinase inhibitors and forms a framework for allosteric-site prediction that can be potentially tested by experiment.

## Methods

### Functional site interaction fingerprint

Functional site interaction fingerprint (Fs-IFP) is a method to determine the functional site binding characteristics and to compare binding sites on a proteome scale [5]. Here we use Fs-IFP to reveal the binding characteristics of the MEK-inhibitor complex for all released MEK structures from the Protein Data Bank (PDB) [39]. In brief, firstly we downloaded all available MEK structures from the PDB; 35 MEK1 structures and 1 MEK2 structure (PDB id 1s9i). The 29 ligand-bound MEK structures formed the MEK structure dataset. We aligned all the binding sites of these ligand-bound structures using SMAP [33–35] and encoded the Fs-IFP as published in an earlier paper [5] using the Pyplif software [40]. Because MEK1 and MEK2 have high sequence identity in their respective kinase domains and their sequence identity is 100% in the allosteric binding site [29,41], in this paper, all residue numbering is relative to the MEK1 sequence. The end result of Fs-IFP calculations for each structure is a one-dimensional bit representation of a variety of interactions between every involved amino acid and the ligand.

### Pharmacophore modeling

Pharmacophore modeling was performed using Maestro from Schrodinger release 2016.02 [42]. Using the MEK structure dataset we found IC50 data for 19 entries (S1 Table). The pharmacophore model was trained using the ligands with an IC50 of less than 10 nM.

### MD simulation

Two MD simulations were performed with starting conformations taken from the PDB: pdb id 4an2 for the MEK-Cobimetinib complex; and pdb id 3zls for the MEK apo structure. Both initial conformations were prepared for MD simulation using the ACEMD protocol [43]. The protonation states of both systems were assigned a pH of 7.0, similar to the cellular environment. Then every His state and every disulfide bond were checked to make sure they conformed to a pH of 7.0. The systems were solvated in a rectangular water box with at least a 12Å shell buffer from any-solute atoms. Charged ions were added to ensure an ionic strength of 0.20 M and electroneutrality. The CHARMM27 force field [44,45], CHARMM general force field [46] and TIP3P force field were used for the kinase, ligand, and water molecules, respectively. Both simulations were relaxed with the standard MD protocol; 2ps minimization, 100ps for NVT, 1ns for NPT with heavy-atom constraints and 1ns for NPT without any constraints. Subsequently, 0.6 μs MD simulations were performed. In both MD simulations all bonds were constrained using SHAKE and the integration time step was 4 fs. The temperature bath used the Langevin method, and 1atm pressure was maintained using the Berendsen method. Both simulations were carried out using the ACEMD software [43] on the NIH high-performance Biowulf cluster (https://hpc.nih.gov/). The MD results were analyzed using the conformations during the last 0.5 μs MD trajectory. The analysis of MD trajectories, including Root Mean Square Deviation (RMSD), Root Mean Square Fluctuation (RMSF) and Principal Component Analysis (PCA), were performed with the Wordom tool [47].

### Screening for similar binding pockets across the human structural kinome

Approximately three thousands protein kinase structures have been solved by X-ray and NMR methods. 2797 of these kinase structures included the catalytic domain and formed the human structural kinome [5]. We then used the MEK-Cobimetinib complex (PDB id 4lmn) as a template to rank similar binding sites from the human structural kinome by performing a one-to-all comparison using SMAP [33–35]. The top-ranked binding sites with *p*-values < 0.05 were retained for further analysis.

### Secondary structure prediction

Determining type-III inhibitors needs to be validated, not only by biological activity assay, but also through analysis of an appropriate crystal structure [8]. Unfortunately, the type-III binding pocket is often not present in the crystal structure when there is no specific type-III inhibitor co-crystalized [8]. To obtain the MEK-similar allosteric site in such kinases, we predicted the MEK-like secondary structure of the activation loop to establish the binding pose of the type-III inhibitor [37]. We used a protein secondary structure prediction server Jpred4 [48] for the task. First, the 516 kinase domain sequences for the eukaryotic protein kinase superfamily were downloaded at kinase.com and were aligned using the cluster omega software [49]. Then, from the alignment, the amino acid sequence of the activation loop for every kinase was extracted with an additional 11 N-terminal and 11 C-terminal residues adjoining the DFG segments. Finally, the activation loop structure was predicted using the JPred RESTful API (v1.5) [48] with default parameters.

## Supporting information

S1 Fig ACP-Cobimetinib-MEK co-crystal structure (pdb id 4an2). a) Cartoon model. b) 2D diagrams of Cobimetinib/MEK interactions including ACP (marked as Acp1383) generated with LigPlot+.
(TIFF)

S2 Fig Cα atom RMSD. (TIFF)

S3 Fig Sequence level similarity for the three kinases BRAF, MEK, and P38α. (TIFF)

S1 Table List of MEK active inhibitors. (PDF)
