# Supplementary figures and images for: Insights into the binding mode of MEK type-III inhibitors. A step towards discovering and designing allosteric kinase inhibitors across the human kinome
Source: PLoS One. 2017 Jun 19;12(6):e0179936. doi: 10.1371/journal.pone.0179936 (PMC5476283; doi:10.1371/journal.pone.0179936)

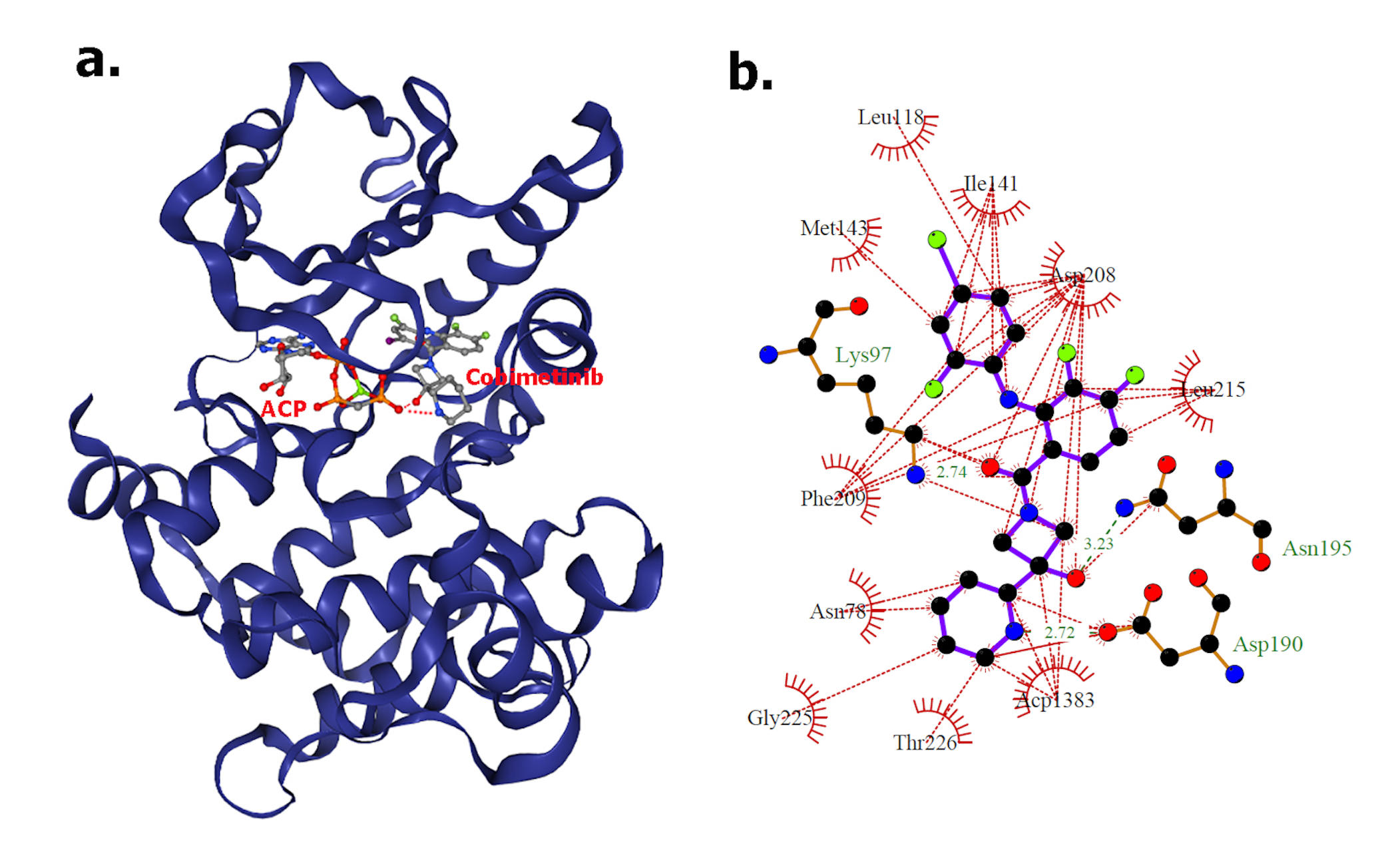

Supplement: S1 Fig — a) Cartoon model. b) 2D diagrams of Cobimetinib/MEK interactions including ACP (marked as Acp1383) generated with LigPlot+. (TIFF) [file pone.0179936.s001.tiff]

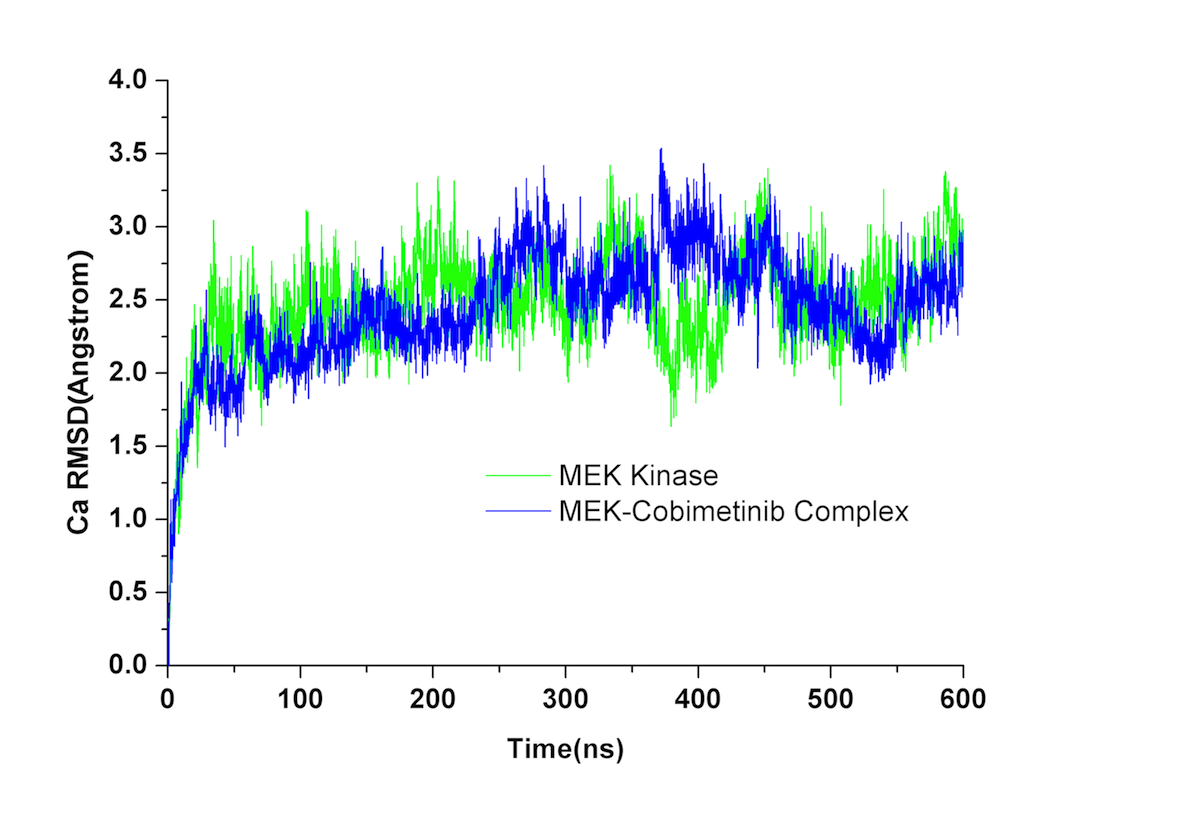

Supplement: S2 Fig — (TIFF) [file pone.0179936.s002.tiff]
