# Supplementary material for: Insights into the binding mode of MEK type-III inhibitors. A step towards discovering and designing allosteric kinase inhibitors across the human kinome
Source: PLoS One. 2017 Jun 19;12(6):e0179936. doi: 10.1371/journal.pone.0179936 (PMC5476283; doi:10.1371/journal.pone.0179936)
Supplement: S1 Table — (PDF) [file pone.0179936.s004.pdf]

S1 Table.

PDB ID	Ligand Name	IC50 (nM)
3DV3	MEK	21
4MNE	573	35
4ARK	M3K	61*
4ANB	YQY	6.2
4AN9	2P7	44
4AN2	EUI	0.9
4AN3	5Y0	0.6*
3V01	3V0	25
3PP1	IZG	3.2
3OS3	3OS	235*
3ORN	3OR	8.8
3MBL	LSG	18.0
3EQH	5BM	60.0
3EQG	4BM	5.2
3E8N	VRA	19.0**
3DY7	1CX	2.0
3V04	V04	13.0
3VVH	4BM	5.2
4LMN	EUI	0.9

* Data from PDBbind; ** data from Binding MOAD; remaining data from bindingDB.
